# Examining seasonal variation in epistaxis in a maritime climate

**DOI:** 10.1186/s40463-019-0395-y

**Published:** 2019-12-30

**Authors:** Ben McMullin, Paul Atkinson, Natasha Larivée, Christopher J. Chin

**Affiliations:** 1 Dalhousie Medicine New Brunswick, Saint John, New Brunswick Canada; 2 Department of Emergency Medicine, Dalhousie University, Horizon Health Network, Saint John, NB Canada; 3 Division of Otolaryngology- Head & Neck Surgery, Department of Surgery, Dalhousie University, Halifax, NS Canada

**Keywords:** Epistaxis, Season, Maritime, Climate, Humidity, Temperature

## Abstract

**Background:**

Epistaxis is a common reason patients present to the emergency department. There is significant variation in climate across Canada. Our study aimed to determine if epistaxis is related to season, temperature and humidity in a Maritime climate.

**Methods:**

Ethics approval was obtained. A retrospective chart review was performed. Patients who presented to the Saint John Regional Hospital Emergency Room between July 2015 and December 2017 with a diagnosis of epistaxis were identified. Weather data was collected from Environment Canada. We performed multiple univariate analyses examining confounding variables.

**Results:**

In total, 476 cases of epistaxis were identified. There was a significant seasonal variation; the highest number of epistaxis cases occurred in the winter (*p* < 0.001). A negative correlation was seen between mean daily humidity and epistaxis (*R*^2^ = 0.7794).

**Conclusion:**

The highest number of cases presented in the winter and a negative correlation was found between epistaxis and mean daily humidity.

## Introduction

Epistaxis is a common occurrence and accounts for 1 in 200 emergency room visits [1]. Sixty percent of individuals will have an episode of epistaxis in their lifetime, and 6% will need medical treatment [2]. The vast majority of epistaxis incidents are anterior in origin, arising from Kiesselbach’s plexus in Little’s area of the nasal septum. In approximately 5–10% of cases, epistaxis originates posteriorly, most often from a branch of the sphenopalatine artery. These bleeds, although less common, are more severe in nature [1].

There is a common belief that epistaxis occurs in the colder, dryer months. Sowerby et al. examined rates of epistaxis in two Western Canadian cities and found a seasonal variance in epistaxis presentations to the Emergency Room in Edmonton, Alberta, but not Calgary, Alberta. In both cities, a negative correlation was found between increasing temperature and epistaxis frequency, but no correlation was noted with humidity [3]. In a study by Reis et al. in Lisbon, Portugal, epistaxis was seen more frequently in the winter months [4].

Saint John, New Brunswick, is a Maritime city in Eastern Canada located on the Bay of Fundy. As a result of this location, there is significant variability in temperature and humidity within the year: the summer months are often relatively humid, while the winter months can be quite dry and cold. As the environmental humidity decreases, it is theorized that the thin mucosa in the nasal cavity is more susceptible to micro-abrasions, thus leading to an increase in epistaxis. This relationship has never been examined in the Maritime climate to our knowledge; therefore, our study aims to evaluate whether this change in climate affects the rate of presentations to the Emergency Department (ED) with epistaxis in a Maritime city. We hypothesize that the presentation of patients with epistaxis to the ED will be higher in the winter months, when the weather is cold and significantly less humid.

## Methods

A retrospective health records review was performed. Research Ethics Board approval was obtained. Using the Canadian Emergency Department Information System (CEDIS), an epistaxis database was constructed including all patients presenting to the Saint John Regional Hospital between July 2015 and December 2017 with a disposition diagnosis of epistaxis. Patient demographics including age, sex, and area of residence were all recorded, as well as presence of a coagulopathy, anti-platelets or anti-thrombolytics use, presence of recent sinus surgery, history of chronic rhinosinusitis, and number of epistaxis episodes in each bleed. Patients presenting with recurrent episodes of epistaxis within a month of each other were recorded as being a single event. The total number of visits to the Saint John Regional Hospital Emergency Department was recorded and used as a baseline.

Weather data was collected from Environment Canada, using readings from the Saint John Airport [5]. The mean daily temperature was recorded, as well as the mean daily humidity value. The seasons were divided as follows: Winter was defined as December, January, and February; Spring was defined as March, April, and May; Summer was defined as June, July, and August; and Fall was defined as September, October, and November.

Descriptive statistics were used to describe demographic and clinical characteristics of all epistaxis cases. Categorical variables were presented as counts with corresponding proportions, and continuous variables were presented as means with standard deviations. Simple t-tests were used to generate *p* values between binomial variables. Chi-square tests with two-way contingency tables were used to generate p values for categorical variables with more than two groups such as seasonal variability and monthly variability. We also used the Marascuillo procedure to calculate individual differences between categorical variables within the seasonal variability analysis.

Finally, mean daily humidity was plotted as a function of number of epistaxis cases. Due to initial data collection constraints, we were unable to perform an analysis that would assess any correlation between humidity and presentation of epistaxis, as many patients would not accurately remember exact symptom onset. Nonetheless, we plotted mean daily humidity as a proxy analysis and applied a line of best fit, as well as a correlation coefficient (R [2]) to the data.

## Results

**Fig. 1:**
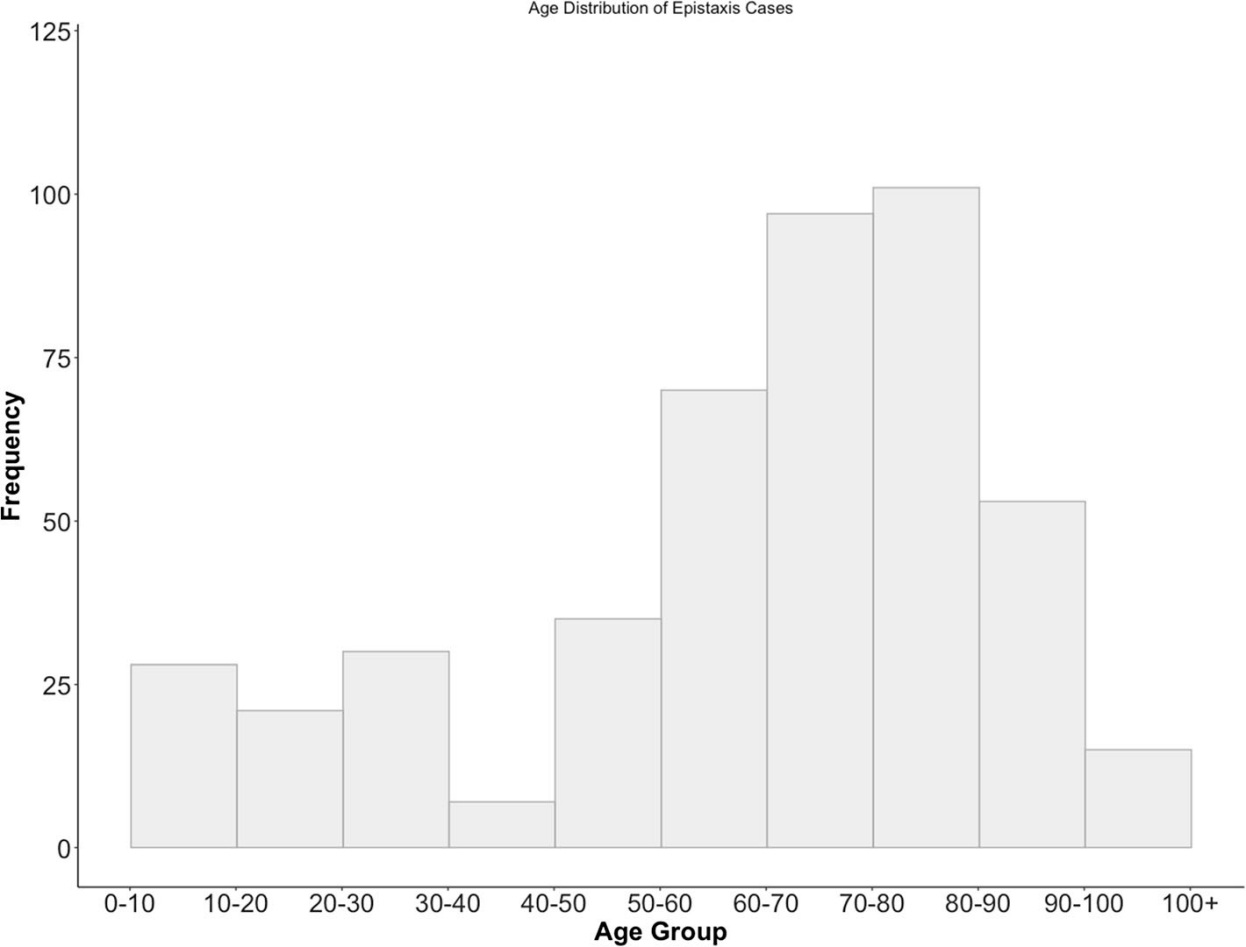
Age-adjusted distribution of patients presenting to the Saint John Regional Hospital Emergency Department with epistaxis

A total of 475 cases were recorded between July 2015 and December 2017 from the Saint John Regional Hospital (SJRH). Data from the CEDIS was available beginning in July 2015, and therefore this is when we began data collection. Epistaxis accounted for 0.3% of all ED visits during this time period. A summary of patient characteristics is shown in Table 1. We observed a bimodal distribution for age in the series of epistaxis cases, with peaks seen at 20–30 years and 70–80 years (Fig. 1).

**Table 1:** Descriptive characteristics of patients presenting to the Saint John Regional Hospital Emergency Department for epistaxis

Variable	N (%) *n* = 475	*P* value
Age (Mean)	59.1	
Sex		
Female	229 (48.2)	> 0.2
Male	246 (51.8)	
Trauma		
Yes	28 (5.9)	*P* < 0.0001
No	444 (93.5)	
Unknown	3 (63.2)	
Anti-thrombolytics/Anti-platelets		
Yes	242 (50.9)	*P* = 0.0520
No	212 (44.6)	
Unknown	21 (4.4)	
Hx of Sinusitis/Surgery		
Yes	27 (5.7)	*P* < 0.0001
No	408 (85.9)	
Unknown	40 (8.4)	
Hx of Epistaxis		
Yes	182 (38.3)	*P* < 0.0001
No	253 (53.3)	
Unknown	40 (8.4)

**Fig. 2:**
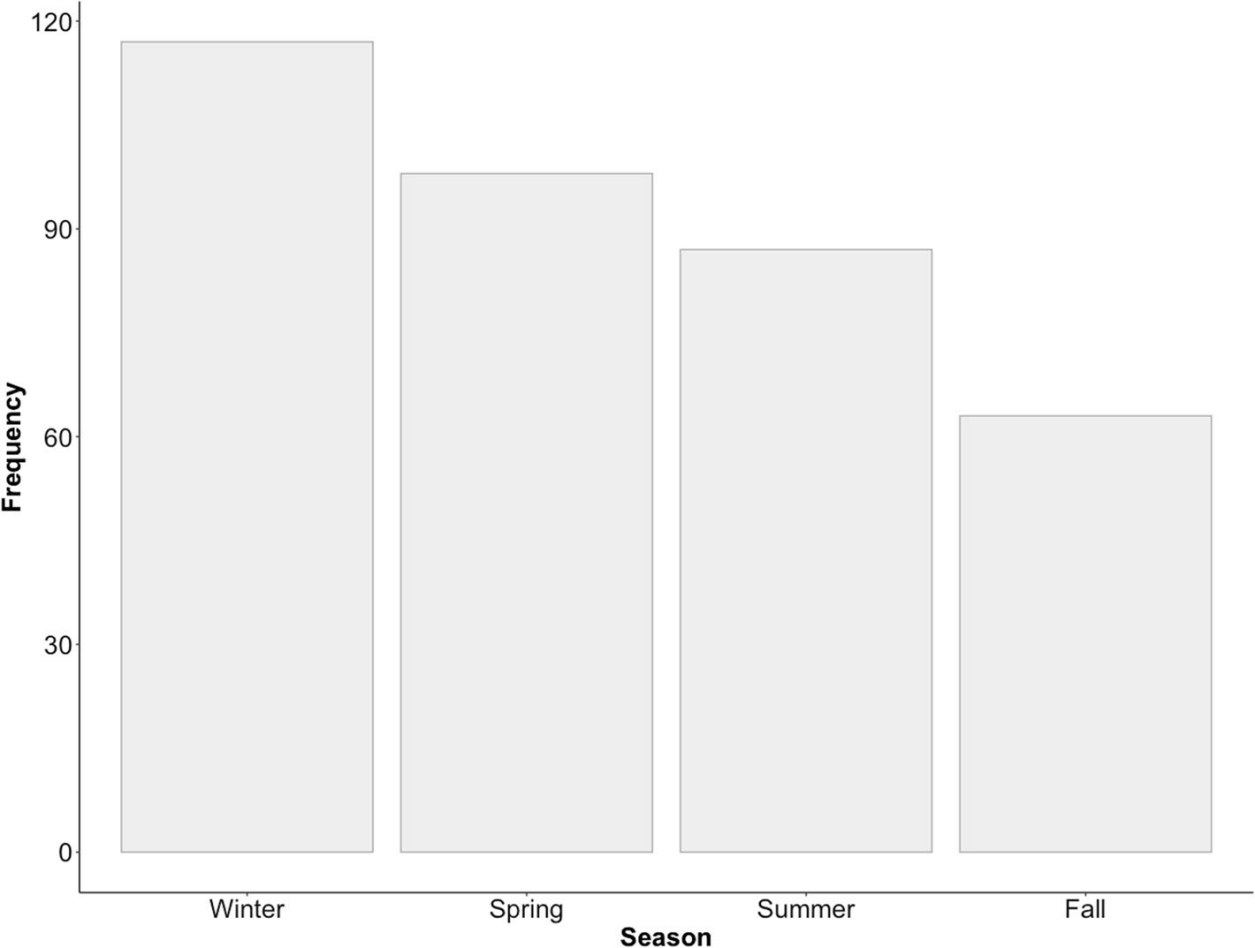
Number of cases of epistaxis presenting to the Saint John Regional Hospital Emergency Department by season between January 2016 and December 2017. The difference between seasons was significantly different (*P* < 0.001)

**Fig. 3:**
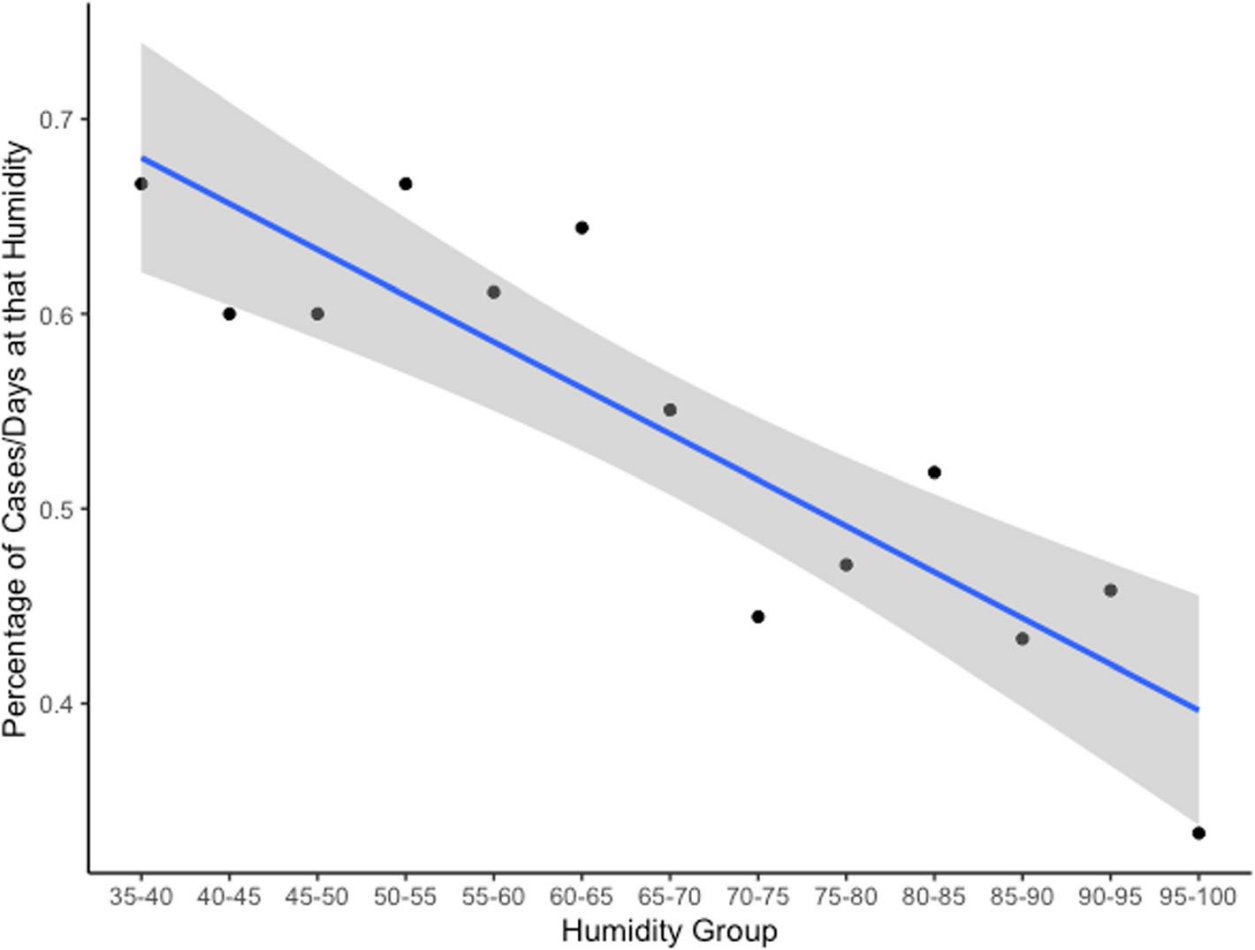
Number of epistaxis cases at each humidity decile, shown from highest humidity decile to lowest. This data is adjusted by the frequency of each humidity decile

The seasonal and monthly data were collected between January 1, 2016 and December 31, 2017; epistaxis data from 2015 was removed to ensure only full calendar years were reported. We then removed all patients with a history of sinus surgery for this analysis (*n* = 11). This left us with a total of 365 cases. The highest incidence of epistaxis was seen in February (12.3%), and the lowest was in September (3.6%) (Table 2). A significant seasonal variation was noted, with the most cases of epistaxis seen in the winter (32.1%), and the least number of cases seen in the fall (17.3%) (*P* < 0.0004) (Fig. 2). There was a negative trend observed between mean daily humidity as a function of number of epistaxis cases (*R*^2^ = 0.7794; Fig. 3).

**Table 2:** Prevalence of Cases of Epistaxis Stratified by Month/Season

Variable	Epistaxis Cases *N* = 365 n (%)	
Season*		
Fall	63 (17.3)	*P* < 0.0004 For seasonal variability
Spring	98 (26.8)	
Summer	87 (23.8)	
Winter	117 (32.1)	
Month		
January	32 (8.8)	
February	45 (12.3)	
March	30 (8.2)	
April	34 (9.3)	
May	34 (9.3)	
June	31 (8.5)	
July	32 (8.8)	
August	24 (6.6)	
September	13 (3.6)	
October	23 (6.3)	
November	27 (7.4)	
December	40 (11.0)

## Discussion

Epistaxis is a common presenting symptom in emergency department patients. Our study found a significant seasonal variation in the number of epistaxis cases presenting to the ED, with the highest number of cases in the winter months, and the lowest number in the fall. This confirms our initial hypothesis. In examining the effect of season, temperature, and humidity on the incidence of epistaxis in Calgary and Edmonton, Sowerby et al. found a seasonal variation in epistaxis cases in Edmonton, but not Calgary. The seasonal variation showed a decrease in epistaxis in the summer months, when compared to the other seasons, instead of an increase in the number of cases in the winter [3]. This differed from our study, where we saw an increase in epistaxis presentation in the winter. Our findings were consistent with other studies examining the relationship between seasonal variation and epistaxis [4, 6–13]. Although the majority of studies support a seasonal variation in the number of epistaxis cases, there remains variability in the literature [14].

In this study, we observed a negative correlation between the number of epistaxis cases presenting to the ED and reported atmospheric humidity levels. There is less literature examining the connection between humidity and epistaxis, and the literature that exists is mixed. In a study by Chaaban et al., an inverse relationship between epistaxis rates and humidity was found, similar to our study [6]. This was supported by a study conducted by Comelli et al., where a weak, but significant correlation between air humidity and epistaxis was found to exist [7]. Interestingly, Sowerby et al. found no correlation between humidity and epistaxis incidence in their study [3]. As mentioned previously, the climate in Saint John, New Brunswick, is typically very humid and warm during the summer, and very dry and cold in the winter. We hypothesized a seasonal variance in epistaxis presentation would therefore be more pronounced in areas with distinct seasonal patterns, which is what was seen in our study.

It is unclear why rates of epistaxis presentations might vary with temperature or humidity, however it is possible that weather changes could be simply an association rather than a true causative factor. For example, it is known that certain upper respiratory infections have an increased prevalence in the winter months, and perhaps the increased inflammation and trauma in the upper airway results in a higher rate of epistaxis [15, 16]. However, the relationship between epistaxis and upper respiratory tract infections would require further examination before conclusions can be made about this.

Our study has some limitations that should be addressed. This study was a retrospective health records review, which is a limitation itself. We also used the mean daily humidity level, but it should be noted that humidity fluctuates and varies throughout the day. The database used to collect the data (CEDIS), only had data from July 2015 to December 2017, which is a relatively short time period. Having a longer time period for this study would have given more validity to its conclusions. This study focused on epistaxis in the ED, and therefore we have missed epistaxis that may be seen in other primary care settings, such as General Practioner offices. This study was also conducted using data from only one center, the Saint John Regional Hospital. Using data from multiple centers would have given us a larger sample size, however, it would be tougher to assess the effect climate has on epistaxis since there is substantial variability in the climate throughout the province of New Brunswick.

## Conclusion

This study examined epistaxis in a maritime climate and found a significant seasonal variation in the presentation of epistaxis with the highest number of cases presenting in the winter months. We also found a negative correlation between the number of epistaxis cases and mean daily humidity.

## Data Availability

The datasets generated during and/or analysed during the current study are available from Environment Canada, http://climate.weather.gc.ca/climate_data/hourly_data_e.html?hlyRange=2012-06-04%7C2019-02-13&dlyRange=2012-06-07%7C2019-02-13&mlyRange=%7C&StationID=50310&Prov=NB&urlExtension=_e.html&searchType=stnName&optLimit=yearRange&StartYear=2015&EndYear=2017&selRowPerPage=25&Line=0&searchMethod=contains&txtStationName=Saint+John&timeframe=1&Year=2016&Month=1&Day=1#.
